# Smart Learning Services Based on Smart Cloud Computing

**DOI:** 10.3390/s110807835

**Published:** 2011-08-09

**Authors:** Svetlana Kim, Su-Mi Song, Yong-Ik Yoon

**Affiliations:** Department of Multimedia Science, Sookmyung Women’s University, Chungpa-Dong 2-Ga, Yongsan-Gu 140-742, Seoul, Korea; E-Mails: xatyna@sm.ac.kr (S.K.); songsm0328@naver.com (S.-M.S.)

**Keywords:** cloud computing, context-awareness, smart learning service, e-learning, ontology

## Abstract

Context-aware technologies can make e-learning services smarter and more efficient since context-aware services are based on the user’s behavior. To add those technologies into existing e-learning services, a service architecture model is needed to transform the existing e-learning environment, which is situation-aware, into the environment that understands context as well. The context-awareness in e-learning may include the awareness of user profile and terminal context. In this paper, we propose a new notion of service that provides context-awareness to smart learning content in a cloud computing environment. We suggest the elastic four smarts (E4S)—smart pull, smart prospect, smart content, and smart push—concept to the cloud services so smart learning services are possible. The E4S focuses on meeting the users’ needs by collecting and analyzing users’ behavior, prospecting future services, building corresponding contents, and delivering the contents through cloud computing environment. Users’ behavior can be collected through mobile devices such as smart phones that have built-in sensors. As results, the proposed smart e-learning model in cloud computing environment provides personalized and customized learning services to its users.

## 1. Introduction

Thanks to the advances in information technologies and high-speed networking, the e-learning environment can offer a new paradigm of learning to learners. Traditionally e-learning offers teaching and learning by wired computers and in a lecture-style classroom setup only. Even though learners were able to browse and download information anytime and anywhere through the existing e-learning platform, they were limited to wired classroom setups.

Smart learning (s-learning) is an important and new paradigm of learning today. The concept of s-learning plays an important role in the creation of an efficient learning environment that offers personalized contents and easy adaptation to current education model. It also provides learners with a convenient communication environment and rich resources. However, the existing-learning infrastructure is still not complete. For example, it does not allocate necessary computing resources for s-learning system dynamically [1,2]. Currently, the majority of s-learning systems have difficulty in interfacing and sharing data with other systems, *i.e*., it falls short of systematic arrangement, digestion and absorption of the learning contents in other systems. This may lead to duplication in creating teaching resources and low utilization of existing resources. To resolve this problem, it is recommended to use cloud computing to support resource management.

The cloud computing environment provides the necessary foundation for the integration of platform and technology. It integrates teaching and research resources distributed over various locations by utilizing existing conditions as much as possible to meet the demands of the teaching and research activities. The cloud computing environment with respect to s-learning offers new ideas and solutions in achieving interoperability among heterogeneous resources and systems. The cloud services mean that the Internet can be used as huge workspace, repository, platform, and infrastructure. Learners can access to the Internet from anywhere at anytime, using widely spread mobile devices but the existing cloud computing technologies are only passively responsive to users’ needs. This situation necessitates proactive cloud services rather than passive services. Since learners typically carry mobile devices of some kind at their hands, the volume of information and services processed through the devices continues to increase.

In this paper, we propose a smart cloud computing (SCC) model for smart learning contents through the E4S—smart pull, smart prospect, smart content and smart push—based on the user behavior acquired by the sensors in the users’ mobile devices. As results, SCC can provide customized contents to each user. Hence users are able to receive customized contents or services they want, without explicit searches. In the subsequent chapters, we briefly describe existing cloud computing services and introduce the proposed context-aware cloud computing environment. Then, we explain the smart cloud service and formally define SCC (Smart Cloud Computing) in detail.

## 2. Related Work

Advances in mobile and wireless technologies have changed the way learners access and share resources, acquire knowledge, and collaborate with each other. Such technologies may include various mobile devices such as hand-held computers and smart phones, embedded sensors in those devices, high-speed wireless networking technologies such as 4G networks that allow those heterogeneous devices to interconnect together. The platform advancements enable context awareness in a smart cloud computing environment and smart services for innovative learning processes. The rest of this section discusses a cloud computing project that uses cloud-based applications in s-learning and an overview of context-awareness in a learning environment.

### 2.1. Cloud Computing and Cloud-Based Applications in S-Learning Environment

The cloud computing is defined as a technology that provides its users with IT resources by using the Internet as a medium. The users can use IT resources such as application software or storage space from the cloud without needing to own them. The users only need to pay per usage charges for the resources they used. The concept of cloud computing is not new. It is the combination of distributed computing, grid computing, utility computing, *etc.* [3–5].

When a user requests services from some cloud server, the server immediately provides the requested services to the user based on their request details. It means that the cloud computing has the ability to customize its service to each user. Since servers charge fees based on usage, it can automatically guide its users of their service request based on previous usages. These features allow that users to use the service only the amount they need at their desired time. Also, there are numerous cloud based applications that are freely available and the trend for that continues to grow [6,7].

There are a number of cloud-based applications available in the e-learning sector as well. Casquero *et al*. [8] presented a framework based on iGoogle and using the Google Apps infrastructure for the development of a network of cooperative personal learning environments. They discussed the integration of institutional and external services in order to provide customized support to faculty members in their daily activities. They also take advantage of the framework as a test-bed for the research, implementation and testing of their educational purpose services. Marenzi *et al.* [9] investigated how educational software can be used in an academic or corporate learning environment. They integrated models and tools that they developed into an open source environment for the creation, storage and exchange of learning objects as well as learning experiences. They presented the “LearnWeb 2.0” infrastructure to support lifelong learning and to enhance the learning experience. This infrastructure brings together information stored on institutional servers, centralized repositories, learners’ desktops, and online community—sharing systems like Flickr and YouTube. Sedayao [10] proposed an online virtual computing lab that offers virtual computers equipped with numerous applications such as Matlab, Maple, SAS, and many others that can be remotely accessed from the Internet.

### 2.2. Context-Awareness in S-Learning Environment

In recent years, context-aware computing has received increasing attention from the mobile learning and cloud learning research communities. Context-awareness is often imposed on intelligent human-machine interfaces that are supported by mobile devices with sensing technologies. The context-awareness is utilized in the interactions between human and their physical environments. In the mobile and ubiquitous computing research field, context-awareness has often been defined as “systems [that] adapt according to the location of use, the collection of nearby people, hosts, and accessible devices, as well as to changes to such things over time” [11]. Context-awareness is about the state of IT devices and their users, including surroundings, situation, and to a lesser extent, location and proposed a 3-D context model with dimensions environment (physical, social), self (device state, physiological, cognitive), and activity (behavior, task) [12].

The s-learning context covers all e-learning situations—from physical settings to virtual space, from individual interests to social culture, from explicit conversations to tacit cognition, from technical media to human emotions, *etc*. The context-awareness for smart learning is defined as the awareness of the whole e-learning context, including awareness of knowledge context, social context and technical context. Context-awareness aims to help learners get an understanding of themselves in the knowledge, social and technical dimensions. It also helps to eliminate or to reduce possible learning obstacles in these aspects, so that more effective learning is possible.

### 2.3. Role of Smart Cloud in Smart Learning

**Figure 1.**
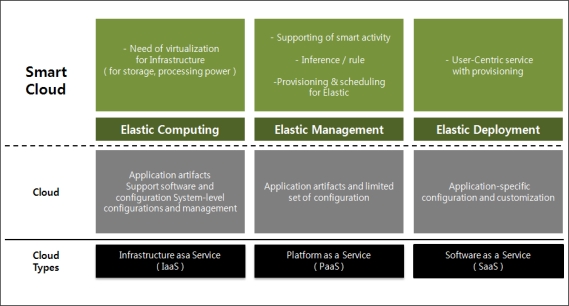
Elastic services in a smart cloud environment.

Smart cloud computing enables cloud servers to provide smart learning services to users through additional intelligent processes on existing cloud systems. The categorization of the demands for smart cloud computing is shown in the Figure 1. The most important responsibility of the servers is to perform elastic processes such as Elastic Computing for Infrastructure as a Service (IaaS), Elastic Management for Platform as a Service (PaaS) and Elastic Deployment for Software as a Service (SaaS) constantly. The elastic processing can be described as collecting user information that is pulled by the sensors in the users’ mobile devices and process the pulled information in real-time so that it can accommodate users’ changing situation dynamically.

To satisfy the requirements of IaaS, such as storage limitation and processing power, the server must be equipped with virtualization capabilities for mass storage and infrastructure in the cloud by incorporating elastic computing, which means that the elastic computing supports dynamic adaptation for the needs of the computing environment. This can be accomplished by a decision policy that can handle users’ changing situations. Elastic Management performs provisioning and scheduling for the decision of smart activities. The provisioning and scheduling are performed through an inference engine that utilizes the rules based on three attributes—an object id for user context, a predicate relationship for user behavior, and a value for thinking. The Elastic Management is utilized in PaaS as a smart activity.

The Elastic Deployment considers the priority of requested services and the elastic results of provisioning. It can also generate user interface configurations, such as personalized views and content rendering for each individual user. These processing tasks may include attachment, execution, and display of the intended contents received in user devices.

## 3. Smart Cloud Computing

**Figure 2.**
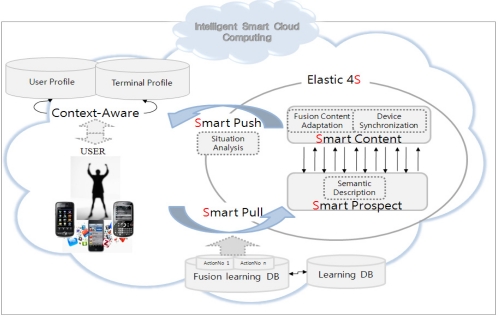
Smart Cloud Computing architecture.

The Smart Cloud Computing (SCC for short) based on elastic computing for 4S model has the capability to provide a smart learning environment. It encourages learning system standardization and provides a means for managing it. A traditional e-learning system can display single content on a single device or multiple contents on one device. The SCC can deliver s-learning to the users so they can use multiple devices to render multi learning contents. The multi learning contents can be played in different devices separately to form a “virtual class”. For this, the SCC uses context-aware sensing. Sensing through the location and IP address of each device, it can orchestrate all devices. The architecture of the model is shown in the Figure 2.

Figure 2 shows how the SCC provides smart learning to the user. It is using Elastic 4S based on information obtained from the user. The information of the user includes the information about the user and the device, received by context-aware sensors. Context-aware monitoring monitors user request(s) and the kind of device(s) that the user is currently using. By using the information collected by the sensors, SCC can provide user-aware service based on Elastic 4S. Elastic 4S is performed through an intelligent learning engine that consists of the rules based on the four services—Smart Pull, Smart Prospect, Smart Content and Smart Push. These four smart services provide high quality services according to the definition of E4S that is described as follows:**Definition 1:** The SCC model for an s-learning service is defined as a set of four tuples.

(1) $$\{E4S_{i}\}=\{(\mathrm{Spul}l_{i},\;\;\mathrm{Spro}s_{i},\;\;\mathrm{Sco}n_{i},\;\;\mathrm{Spus}h_{i})\},\;\;1\leq i\leq N$$

where:

*Spull_i_*: Smart pull—analyze the extractable content from the sensing information.
*Spros_i_*: Smart prospect—description of the content for target devices and delivery time.
*Scon_i_*: Smart content—connection establishment between server and target devices.
*Spush_i_*: Smart push—synchronized delivery of contents to target devices.

As shown in the definition, the E4S pulls the sensing data and analyzes the extractable contents. The context-aware module is functioning as an information filter that extracts only the intended information from the sensing data. There can be multiple contexts in sensing data depending on the services available in the learning management system. They are individually synchronized and pushed by the smart learning service. The sensing data analysis process identifies the different backgrounds of each learner and accommodates each learner’s needs individually. Learning contents are customized based on the background and learning needs. It means that different learning content may have unique technical and functional characteristics and may use different communication channels. Each customized learning contents may differ in modality (*i.e*., text-based, audio, video, *etc*.), capability (*i.e*., bandwidth), and timing (*i.e*., types of synchronization).

## 4. The SCC

### 4.1. Context-Aware Module

To provide the smart learning service to each individual user, the context-aware module must automatically deduce the actual situation from the user’s behavior. The context-aware module as shown in the Table 1 presents the context model based on a hybrid situation that consists of the user situation and the physical situation. The context model includes static factors and dynamic factors that describe the hybrid situation. The context model deals with the context objects and the relations among them. Since the context-aware module considers the characteristics of each user individually, such as learners’ knowledge interests, needs, expertise, and experiences, it can provide highly customized and relevant learning services to each user.

The user situation contains the detailed information about users. The user preferences in the user situation specify user actions and required services. A user action indicates some preference in user’s requests for learning services. The required service should help users acquire knowledge in the area of interest, share experience, and collaborate with each other in learning. Each user’s personal information such as personal context is secured by some security setting such as user’s schedule and location.

The physical situation includes each terminal’s MAC address, capability, software interface status, and types of software applications. The terminal capability describes the process speed, memory, screen size, resolution, and interface types. The terminal application type describes software applications installed in the terminal. The application type is based on quality of service (QoS) parameters, such as response time, delay, jitters and bandwidth. It can be categorized into four types, namely (1) conversational service, such as VoIP; (2) real-time (RT) service such as Internet Protocol Television (IPTV) and mobile TV; (3) non-real-time (NRT) services such as email or ftp; (4) interactive services such as web browsing. The context model has user situation information such as user’s requests and the devices they are using. Using this information, the SCC can provide user-aware smart learning service based on E4S. The E4S handles the pulling of sensing information, the analysis of context from the pulled information, the generation of smart content, and the push of smart learning service to individual terminals with different contexts.

### 4.2. The Elastic 4S (E4S) System

In this section, we discuss the E4S—Smart Pull, Smart Prospect, Smart Content, and Smart Push—in detail.

#### 4.2.1. Smart Pull

The Smart Pull is the process of extracting learning content in the fusion learning DB through a variety of sensors from the user’s device including user situation and technical situation information based on the context model. The context model is used to filter information from the sensing information. The filtered information is analyzed to determine user’s behavior patterns.

**Figure 3.**
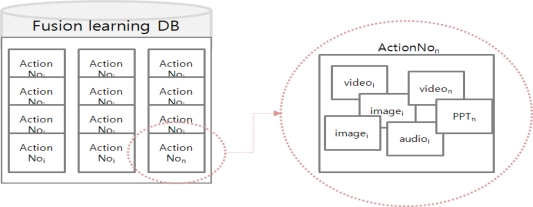
ActionNo in Fusion learning DB.

The Smart Pull identifies a right action service in fusion learning DB based on the user action in context model. The fusion learning DB consists of various multimedia learning materials such as video, text, PPT and image scattered in different learning DB. For example, if a user action in the context model requests the topic of “multimedia”, the Smart Pull extracts a related action of “multimedia” in fusion learning DB. The fusion contents have some information for teaching with one action scenario (ActionNo) as shown in the Figure 3.

**Figure 4.**
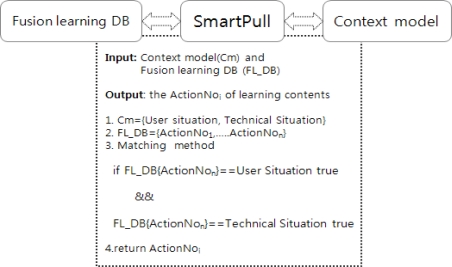
Matching search of Smart Pull.

Figure 4 shows a Smart Pull method for extracting an action scenario (ActionNo) based on a user request action in the context model. For this, the Smart Pull checks the user situation and technical situation information to obtain a correct set of values of the requested learning. The set consists of the user’s learning request action, learning interests, needs, and the application type currently in use. The Smart Pull causes a matching search of learning object in the Fusion learning DB. The matching search uses two conditions to find the right ActionNo in the DB. First, the ActionNo object is the mapping to user action object. Second, the ActionNo content type is the mapping to the running application type in the technical situation. It may occur when a user uses multiple devices. If an ActionNo satisfies both conditions, the ActionNo is sent to the next step for synchronization.

### 4.2.2. Smart Prospect

The Smart Prospect is mainly responsible for describing the contents in ActionNo—time, memory, resolution and supported application types. The description is needed for fusion content delivery, because ActionNo specifies the fusion learning content in the Fusion learning DB. For the delivery of fusion content to a user’s device, the SCC is required for harmony adaptation. In the harmony adaptation, the most important part is synchronization. The synchronization part controls the time for synchronization among fusion contents in the same ActionNo. To access the information such as memory, resolution, or application type of the contents in ActionNo, the Smart Prospect uses a Semantic Description using of UVA (Universal Video Adaptation) model that has been developed by Yoon [13]. The UVA model uses the video content description in MPEG-7 standard and MPEG-21 multimedia framework.

**Figure 5.**
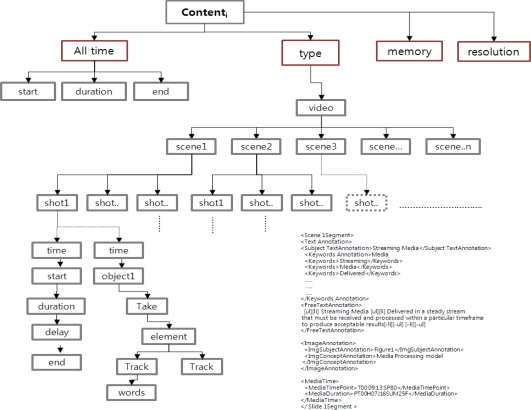
Video analysis based Semantic Description.

The Semantic Description based on the UVA model includes the effort to build a new architecture that supports content with formal semantics. The semantic description provides the accurate and meaningful information for the fusion content. The semantic description uses XML, ontology and Resource Description Framework (RDF) that help define fusion content clearly and precisely. It also represents systematic information about the contents. The role of the ontology is to formally describe the shared meaning of vocabulary used. The ontology describes the basic fusion learning contents of some domain where learning takes place (e.g., history of science). It includes the relations between these concepts and some basic properties. Based on the ontology, all learning content in the ActionNo are associated each other. For example, the description of the video content used in semantic description can be related to the scenes of video as shown in Figure 5.

Figure 5 shows a result of a detailed description of video content in ActionNo based on the UVA semantic description model. The Semantic description provides the detail information about video content for the Smart Prospect. The detailed description consists of time, memory, resolution and supported application types for each content. The time information has two types—<All time> and <time offset>. The <All time> represents playback time of the entire video content. The <time> represents the playback time of a shot in the video content. Based on the two types of time information, the next step—Smart Content—performs a synchronization process that utilizes three elements—<type>, <memory>, and <resolution>. The <type> indicates supporting types for some application, such as video, image, PPT or text. The <memory> and <resolution> specifies memory and resolution information of video content, respectively. The process of synchronization between device and content is accomplished by using the <type, memory, resolution> information.

#### 4.2.3. Smart Content

**Figure 6.**
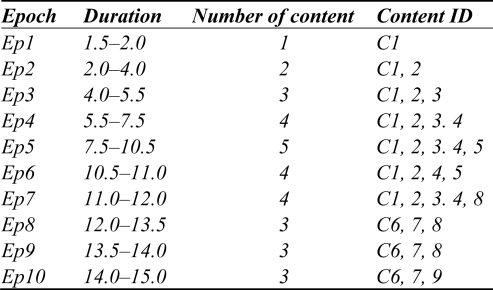

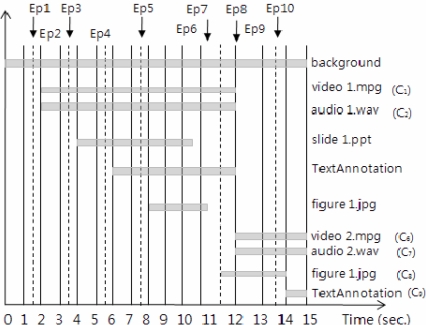
Synchronization of fusion contents.

The Smart Content generates the fusion content for the user’s device using the harmony adaptation. The harmony adaptation has two steps—Fusion Content Adaptation and Device Synchronization process. The Fusion Content Adaptation presents the synchronization among the fusion contents in ActionNo. The Device Synchronization performs the process of synchronization between devices. For the synchronization of the fusion contents, the Fusion Content Adaptation (FCA) uses the contents that are indicated by Semantic description from Smart Prospect. The adapted contents include <All time {start, duration, delay, end}> and <time {start, duration, delay, end}> information. For the time synchronization, the FCA uses Interpreter Playout Schedule (IPS) to schedule the order of playout. The IPS uses the duration time of the adapted contents with consideration of the synchronization among connected devices. The simulation results for sample IPS are illustrated in Figure 6.

According to the IPS, every media stream can be modeled as an agent, or alternatively, any number of the same media type can present a scenario sequence that controls different media streams at different times.

For the process of synchronization between multiple devices, the Device Synchronization creates a channel for each device based on the technical situation in the context model. Each channel includes the unique information for the associated device. The major role of channel management is the synchronization of the channels to communicate with each other when multiple devices are playing. It means that the fusion contents can be played on multi devices at the same time, so it is required to have the coordination among them. The Device Synchronization uses SyncML (Synchronization Markup Language) to set the synchronization between devices. The SyncML is an international standard language for matching data between different devices and applications at any network company (network Ltd.). Through the synchronization between devices, the adapted contents can be used in Smart Push step.

#### 4.2.4. Smart Push

The last step of E4S is the Smart Push process. The Smart Push delivers the smart learning service for user after the fusion contents are adapted and device and content are synchronized. As for the content delivery, the situation analyzer will be used. The situation analysis links the contents and devices so learners can use the learning contents at their device. The situation analyzer uses the physical situation information to find the related details of the contents and devices. The related details for the contents are time, memory, resolution and application types for the contents. The related details for device are process speed, memory, screen size, resolution and supported interface types for the terminal. The details of terminal indicate the information about available resources in the current device. If the details of a device and contents are of the same or compatible type, a link can be established and the contents are delivered to the device.

After a device and contents are synchronized by the situation analyzer, the Smart Push delivers the smart learning service to its users. The Smart Push delivers a complete set of smart contents to the user using terminal’s AP (Access Point) and MAC-Address in the context model of physical situation. The access point (AP) recognizes the physical location area of mobile devices that enter to the location range of AP. The AP assigns an IP-address to a mobile device based on its MAC-Address. The mobile device is connected to the Internet via AP, so we can get the physical location information when a mobile device gets its IP-address. The AP usually connects to a router (via a wired network), and can relay data between the wireless devices (such as computers or printers) and wired devices on the network. The Table 2 shows the context model of physical situation.

**Figure 7.**
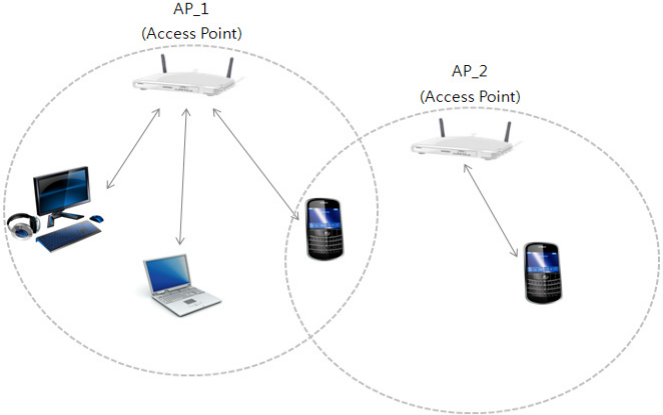
AP as central unit in All-Wireless Network.

Table 2 shows the information of the access point (AP) based on the user’s location. According to the user’s location, the AP handles multiple devices that are within the range of the AP. An access point connected directly to a wired LAN provides a connection point for wireless users. If more than one access point is connected to the LAN, users can roam from one area of a facility to another without losing their connection to the network. As users move out of range of one AP, they automatically connect to the network (associate) through another AP. The roaming process is seamless and transparent to the user. The Figure 7 shows an access point in an all-wireless network.

The AP’s generic information describes the status of the network connection, such as connected or disconnected. As shown in the Table 2, we can know a position of the user from the location area of AP-1 and AP-2. It is because APs configures a MAC (Medium Access Control)-Address and IP-address within its location range. For example of Table 2, the three user devices—MAC addresses are 00:11:93:0d:a7:f5, 00:11:93:8d:f1:50 and 00:11:93:25:t4:bb—are located in physical range of AP-1. However, the AP 2 recognized one device with one MAC-Address. This means that mobile device (MAC-Address is 00:1b:93:e8:c4:1f) did not connected since the mobile device located out-of-range for AP 2. To conserve and effectively manage the resource of the devices, the AP performs the AP Controller. The AP Controller manages the MAC-Addresses for transmitting the learning contents to the linked devices according to the context model.

## 5. Implementation

**Figure 8.**
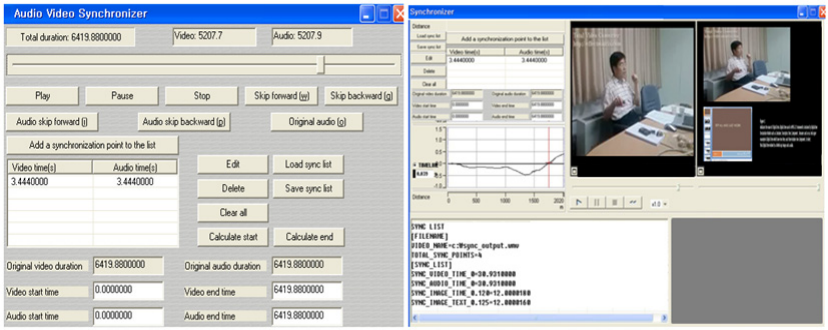
Synchronization of Fusion contents.

As a part of the proof of the proposed concept, we have implemented the fusion media generator. The Figure 8 shows screen shots for fusion content generation and synchronization based on the fusion content adaptation step for an s-learning service. When a list of synchronized fusion-media is uploaded, they can be played in the preview.

The left side of the Figure 8 shows a screen shot of the audio and video synchronizer. The audio and video should have the same duration schedule—start, duration, delay and end time. After synchronization of the video and audio contents is completed, other fusion media (PPT, image and text) may be synchronized according to the duration schedule. The right side of the figure shows the action of synchronized learning contents based on the IPS. The graph in the figure shows the progress of the synchronization. The upper left part shows a synchronization of the video and audio. The part below shows the synchronization with other fusion media. The synchronized fusion media can be played in the preview. In the bottom side of the figure shows the start and duration time of the schedule for the fusion media that are displayed on the screen. Currently the figure shows four fusion media—video, audio, PPT and text. Accordingly, the time schedule of the fusion media is displayed in four lines.

To transfer the fusion media to the multiple devices, the SCC uses the Smart Push process. This process identifies the type of contents being transmitted, determines the order of media, and synchronizes media streams from different sources. The Smart Push may be applied more efficiently in many other multimedia applications, such as video conferencing.

**Figure 9.**
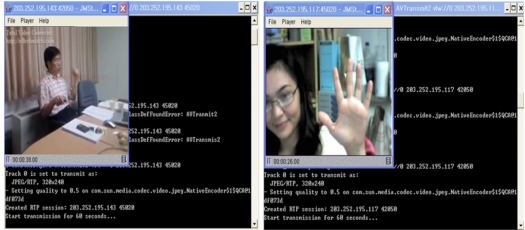
Fusion media being transferred to multiple devices.

In the implementation, we have delivered the synchronized fusion media to two different devices that have different MAC-Addresses—00:11:93:0d:a7:f5 and 00:11:93:8d:f1:50. The AP controller transmits the fusion media to the devices, as shown in Figure 9.

## 6. Conclusions

Modern learning services typically deal with multi-media resources such as graphics, video, images, text *etc*., since such resources provide an efficient learning environment that helps learners understand the topic of interest better. The awareness of user behavior in the learning process can be very helpful in providing the right contents at the right time. The learning services that include the concept of such awareness and the capability of handling multi-media resources efficiently can be termed smart learning systems. In this paper, we have introduced the use of context-awareness for user behavior and a way to deliver the corresponding contents to the users.

The concept of the context model in context-awareness was introduced, which includes the static and dynamic descriptions of the user and physical situation. The context model deals with the context objects and the relations among them. The results of the context-awareness allow learning efficiency and outcomes for smart learning, such as learners’ knowledge interests, needs, expertise, and experiences. Using the context model, the smart cloud model (SCC) can provide the necessary contents to users precisely. In order to collect user’s behavior, sensors in users’ devices were used. Based on the sensing information, the cloud computing environment can forecast and prepare the contents by analyzing the collected information. Such a process enables smart learning services to provide the contents to the users at an appropriate time.

In order to deliver such customized contents to the users at right time, the SCC followed Elastic 4S—Smart-Pull, Smart-Push, Smart-Prospect and Smart-Contents. All the services are based on the collected data through the sensors in user’s device. We have utilized the E4S model and analyzed the sensed information within the category of context. The context-aware model handles the fusion media adaptation, synchronization, and transmission for a smart learning service. We have considered various requirements that for the users, the networks, and the cloud. As a future work, the protocols for smart cloud computing and domain specific ontology will be investigated.

## Figures and Tables

**Table 1.** Context model based on Hybrid Situation.

**Static**	**Dynamic**
*User Situation*	
- User identity	- Schedule
- User preference	- Location
• User action	- Learning request(Learning object, title)
• Required service	- Interest, needs, expertise and experiences
*Physical Situation*	
- Terminal Mac-ID	- Interface status
- Capability	- Running application type
• Process speed	
• Memory	
• Screen size	
• Resolution	
• Supported interface types	
- Application type	
• VoIP	
• IPTV, mobile TV	
• Email, ftp	
• Web browsing	

**Table 2.** Context model of physical situation.

**AP**	**MAC-ID**	**State**	**Memory**	**Resolution**	**Screen size**	**Proc. Speed (Mbit/s)**
1	00:11:93:0d:a7:f5	Connected	4 GB	2,560 × 1,440	27”	216
1	00:11:93:8d:f1:50	Connected	4 GB	2,560 × 1,440	22”	144
1	00:11:93:25:t4:bb	Connected	1 GB	240 × 120	5”	144
2	00:1b:93:e8:c4:1f	Disconnected	1 GB	480 × 360	7.5”	144
2	00:11:93:s6:25:aa	Connected	4 GB	2,560 × 1,440	22”	144
